# Late-onset rupture of an intracranial dermoid cyst: a case report

**DOI:** 10.1186/s13256-023-04322-0

**Published:** 2024-01-08

**Authors:** Richard Shalmiyev, Adam Devine, Sheyla Gonzalez, Mauricio Danckers

**Affiliations:** 1 Department of Medicine, HCA Florida Aventura Hospital, 20900 Biscayne Blvd, Aventura, FL 33180 USA; 2 Division of Pulmonary and Critical Care Medicine, HCA Florida Aventura Hospital, 20900 Biscayne Blvd, Aventura, FL 33180 USA

**Keywords:** Dermoid cyst, Critical care, Neurosurgery

## Abstract

**Background:**

Dermoid cysts are developmental abnormalities occurring between the third and fifth week of embryogenesis. These lesions can initially develop as intracranial or extracranial and persist throughout the patient’s lifetime. While generally benign, their symptoms can be due to mass effect or local irritation secondary to rupture and release of contents, typically presenting as headaches and seizures. Intracranial dermoid cysts are rare and comprise less than 1% of all intracranial lesions, with rupture occurring approximately 0.18% of the time.

**Case presentation:**

Our case describes a 42-year-old Hispanic female with a late-onset rupture of an intracranial dermoid cyst with associated new onset seizures. She underwent uncomplicated neurosurgical resection with mesh placement and was scheduled to follow-up as an outpatient.

**Conclusion:**

To avoid rupture and associated sequelae in future patients, we recommend considering a more invasive approach as the initial strategy if internal cysts are relatively accessible.

## Background

Dermoid cysts are developmental abnormalities occurring between the third and fifth week of embryogenesis. They are generally benign, slow-growing cranial lesions, typically consisting of hair follicles, sweat glands, or sebaceous glands. While these lesions can initially develop intra- or extracranially, intracranial dermoid cysts are rare and comprise  ~< 1% of all intracranial lesions, typically presenting in the first four decades of life [1–6]. Symptoms are primarily due to mass effect or local irritation, most commonly headaches or seizures. A feared complication is the rupture of these cysts, leading to seizures, sensory or motor deficits, chemical meningitis, vasospasm, cerebral ischemia, and death due to the dissemination of its contents into the surrounding space [2, 4, 7–13]. Evidence is scant; however, one neurosurgical case series reported ruptured intracranial dermoid cysts represented approximately 0.18% of all central nervous system tumors surgically treated over a 12 year period [14, 15]. Our case describes a patient with late-onset rupture of an intracranial dermoid cyst with associated new-onset seizures.

## Case presentation

**Fig. 1:**
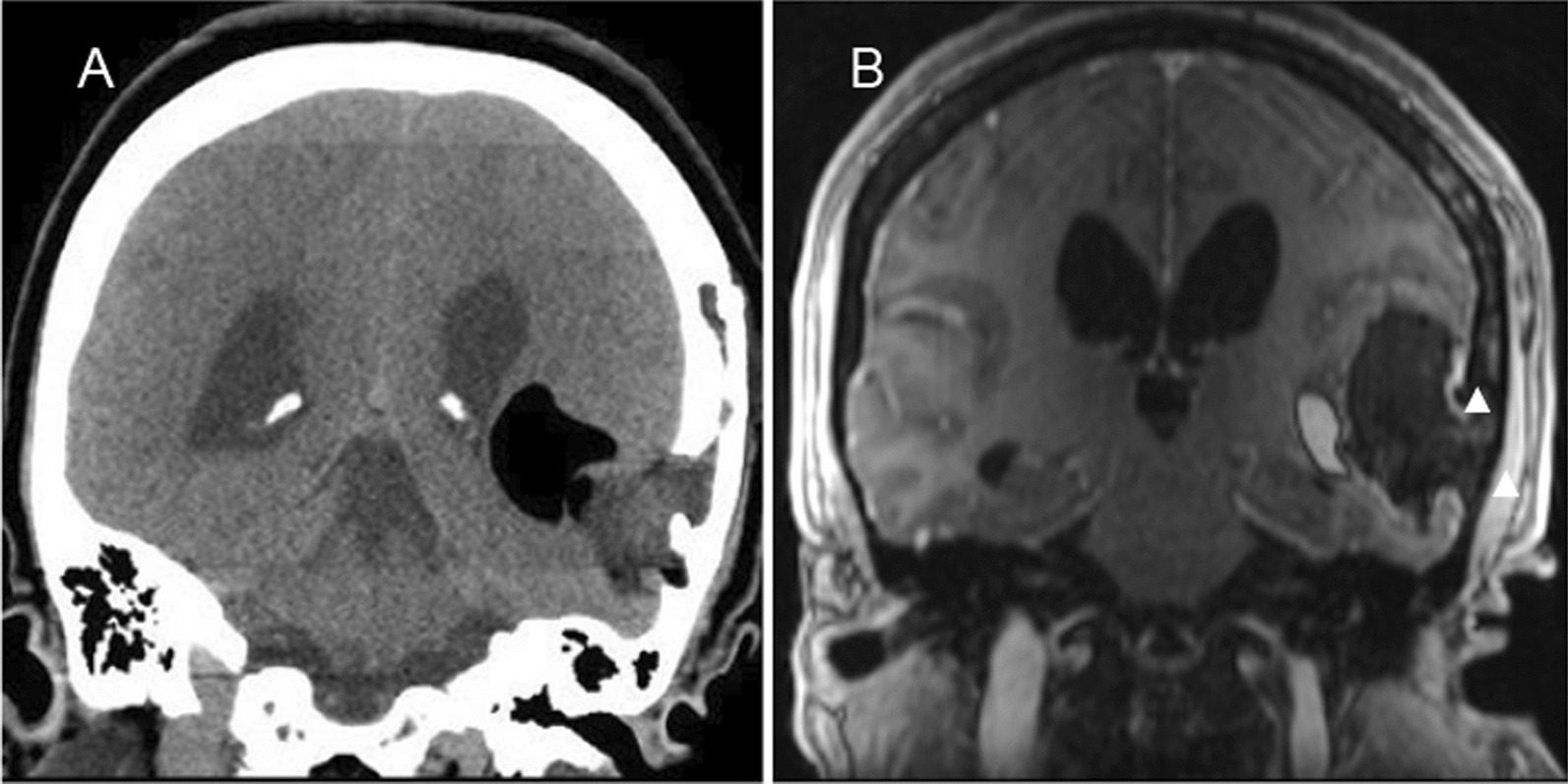
**A** Computed tomography of head. Left-sided fat-density tumor with erosion into the bone and the presence of a fistulous sinus tract. **B** Brain magnetic resonance imaging—T1 flair post contrast. Left temporal mass with subcutaneous extension and tiny fat droplets (arrowheads) in the subarachnoid space

A 42-year-old Hispanic female with a past medical history of a left-sided temporal mass of unknown significance presented to the emergency department after the new onset of a witnessed tonic–clonic seizure. Her seizures ceased after administration of a total of 4 mg midazolam intravenous. She required endotracheal intubation for airway protection. She had carried this temporal cystic subcutaneous mass since adolescence with no intervention at that time. Initial computer tomography (CT) of the brain (Fig. 1A) and magnetic resonance imaging (MRI) of the brain (Fig. 1B) revealed a left frontotemporal, fat-density tumor associated with bone scalloping and suspected partial rupture into surrounding subarachnoid space, with hyperintensities likely representing associated fat droplets.

**Fig. 2:**
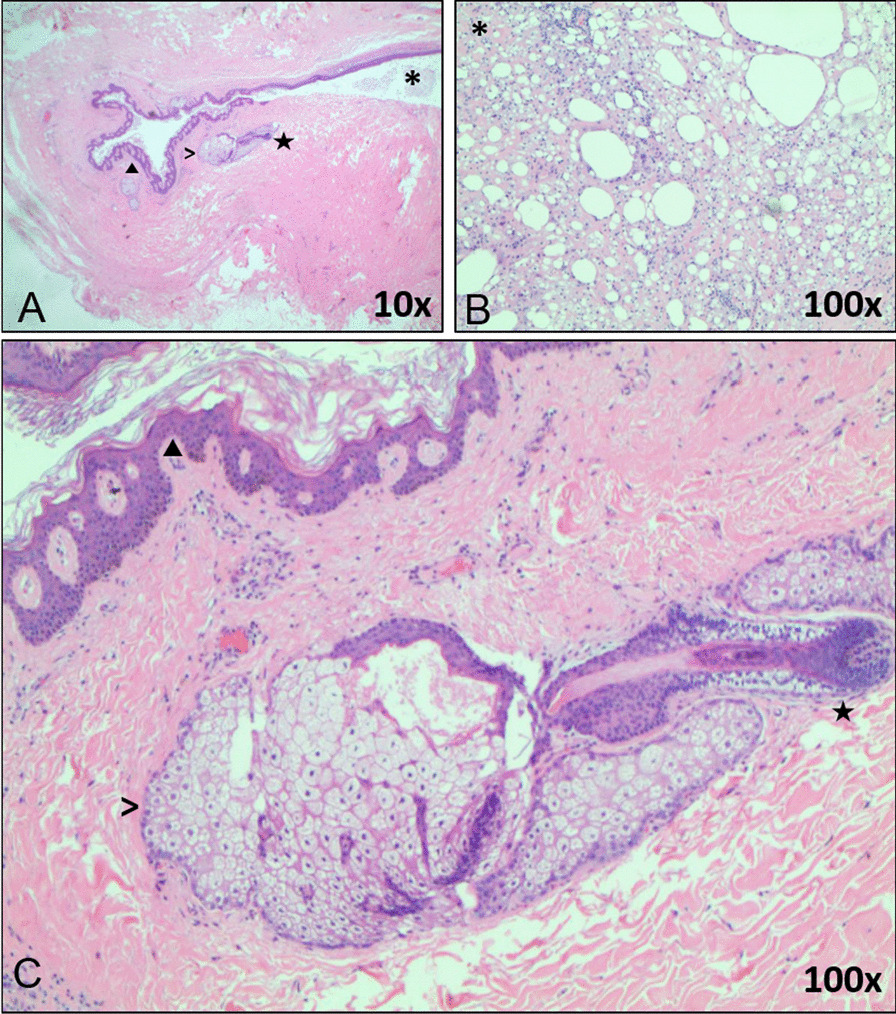
Histopathologic slides using H&E staining.** A** Four tissues at 10× magnification. **B, C** Image **A** at 100× magnification. (Filled triangle) keratin; (>) sebaceous gland; (asterisk) fat necrosis; hair follicle

She subsequently underwent left-sided craniotomy and craniectomy for the resection of two separate masses with titanium mesh cranioplasty the following day. Intraoperative findings revealed two separate tumors eroding through the skull with unclear, intracranial, or extracranial origin. The posterior tumor was completely resected. The anterior tumor was completely debulked; however, only partially resected as a portion of the tumor capsule was adherent to the brain tissue and bled on a further removal attempt due to its involvement with the vein of Labbe and several branches of the middle cerebral artery (MCA). Pathology of the masses was consistent with rupture of a mature teratoma/dermoid cyst and associated fat necrosis, and with multiple tissues present including hair follicles, sebaceous glands, and keratin (Fig. 2). She was discharged on day 7 on a prednisone taper. She was unfortunately lost in follow-up.

## Discussion

Dermoid cysts are developmental abnormalities occurring between the third and fifth week of embryogenesis. The atypical migration and collection of ectodermally committed cells, such as hair follicles, sweat glands, and sebaceous glands, at the time of neural tube closure results in a mucocystic mass in an abnormal location [1, 9, 16]. These lesions can initially develop intra- or extracranially, and generally remain benign and slow growing due to their physiologically active internal dermal elements [1–4]. Intracranial dermoid cysts (IDCs) are rare and comprise ~ < 1% of all intracranial lesions, typically presenting in the first four decades of life [4–6]. The most common locations for IDCs are midline in the posterior fossa, midline/para sellar, frontal, and intradural regions [1, 9, 17]. While largely asymptomatic, patients may experience symptoms due to mass effect or irritation, including headaches, seizures, olfactory hallucinations, cranial nerve compression, and hydrocephalus [6, 17]. Literature shows symptoms generally arise between the ages of 16–60 years, with a mean age of 32 years [6, 18]. Spontaneous rupture of these cysts, while rare, can result in serious clinical events such as headaches, seizures, damage to neurovascular structures, sensory or motor deficits, chemical meningitis, vasospasm, cerebral ischemia, and death due to the dissemination of its contents into the surrounding space [2, 4, 7–13]. The exact pathophysiology of IDC rupture remains poorly understood. Hypotheses include glandular secretions due to age-dependent hormones in adolescent patients, spontaneous rupture, trauma, surgery, and head movements [1, 9, 10, 16]. These contents can seed the intracranial cavity, some unseen by imaging, and cause further complications, predisposing the patient to recurrence [19–21].

Extracranial dermoid cysts (EDCs), sometimes referred to as scalp dermoids, contain same contents as IDCs, but develop extracranially in childhood, with an incidence of  ~20% [3]. While also mostly asymptomatic, they may present as a subcutaneous nonpainful mass. Treatment of choice for EDCs is complete surgical excision. Unaddressed, these cysts have a risk of bony erosion with intracranial expansion, predisposing the patient to similar risk as IDCs. Intracranial dermoid cysts have been shown to develop subcutaneous sinus tracts and extend to the subcutaneous space, which would present the same way on imaging as extracranial dermoid cysts infiltrating the intracranial space [4, 22]. The patient presented in her fifth decade of life, making it difficult to determine what the original mass was. We infer that, due to the long course of her disease and late onset of complications, the initial lesion was an EDC with intracranial expansion ultimately resulting in rupture. Her brain imaging findings, although classic for both IDCs and EDCs, could not fully allow for classification [11, 14]. The neurosurgeon was unable to identify whether the cysts originated intra- or extracranially. Current recommendations regarding the treatment of intracranial dermoid cysts, unlike extracranial dermoid cysts, is conservative management until the patient becomes symptomatic [11]. This, however, poses its own risk to the patient. Untreated, IDCs can exhibit malignant transformation, hydrocephalus, development of a cutaneous sinus tract, recurrent bacterial meningitis, or ultimately rupture [21, 23, 24].

The patient’s rupture seems to have occurred spontaneously as there was no identifiable cause. Furthermore, we suspect that the rupture and content dissemination was the cause of the seizures. While our patient presented with classic symptoms of seizures following a rupture, our case is unique because her only prior sign or symptom was the cystic mass as a child, denying previous loss of consciousness, loss of vision, focal neurological deficits, and seizures. She was initially evaluated at 15 years of age for a fluctuant mass on the left temple, asymptomatic at that time, manifesting over 10 years later than the average age of presentation. Furthermore, if we consider this initial mass to be an EDC, it had an approximately 37–57% for intracranial extension, predisposing the patient to the same aforementioned complications [25]. One critical sequelae to consider is malignant transformation. Even though malignant transformation is a rare complication without a truly known incidence rate, these patients have a poor prognosis, with 60 months postdiagnosis being the longest reported survival time [24]. The pathology shows no signs of malignancy; however, malignancy of the remaining tissue and future development cannot be excluded. She will require close long-term follow-up.

The strength of this case report includes reinforcing the effectiveness of neurosurgical evacuation of dermoid cysts, even in late stages. This raises a possible indication for earlier neurosurgical intervention to prevent similar presentations to this patient. Limitations include the unclear patient history regarding the nature of her initial mass and our loss to follow-up post intervention. However, given the pathology results and initial presentation, her case is consistent with dermoid cyst rupture, allowing us to draw appropriate conclusions.

## Conclusions

Complete removal of EDCs and particularly IDCs can prevent regrowth and morbidity if resected without injury to surrounding neurovascular structure [18, 26–30]. Surgical excision is often reserved until symptoms arise, predisposing the patient to rupture. Had she undergone removal prior to rupture, her subsequent complications may have been avoided. To avoid rupture and associated sequelae in future patients, we recommend considering a more invasive approach as the initial strategy if internal cysts are located on the periphery and relatively accessible.

## Data Availability

All data were accessed through the PubMed search index.
